# A New Environmentally-Friendly System for Extracting Cellulose from Corn Straw: The Low Temperature Laccase System

**DOI:** 10.3390/ma13020437

**Published:** 2020-01-16

**Authors:** Heming Song, Hongge Jia, Qingji Wang, Xinyi Zhao, Guoxing Yang, Mingyu Zhang, Hailiang Zhou, Shuangping Xu, Yu Zang, Yazhen Wang, Liqun Ma

**Affiliations:** 1 College of Materials Science and Engineering, heilongjiang province Key Laboratory of Polymeric Composition, Qiqihar University; Wenhua Street 42, Qiqihar 161006, China; songheming0603@outlook.com (H.S.); zhaoxinyi1119@126.com (X.Z.); zhangmingyuno1@163.com (M.Z.); zhouhailiang95@163.com (H.Z.); xshp_1979_1999@163.com (S.X.); zangyu.25@163.com (Y.Z.); wyz166@163.com (Y.W.); 2 Daqing Oilfield Construction design and Research Institute, XiLing road 32, Daqing 1637241, China; wangqingji@petrochina.com.cn; 3 Daqing Petrochemical Research Center, Petrochemical Research Institute, China National Petroleum Corporation, Chengxiang Road 2, Daqing 163714, China; ygx459@petrochina.com.cn

**Keywords:** cellulose, corn straw, low temperature, laccase

## Abstract

Corn straw is an agricultural waste. The system for extracting cellulose from corn straw at a high temperature has been widely reported by researchers. However, the system for extracting cellulose from corn straw at a low temperature has been rarely reported. In this paper, a new system for extracting cellulose from corn straw at a low temperature was reported for the first time. This new system is designated as the low temperature laccase system (LTLS). Cellulose was successfully extracted from corn straw by the LTLS, and the used solution could be recycled. Therefore, the low temperature laccase system is an environmentally-friendly system. The cellulose content in corn straw is 30–40%. The yield of cellulose extracted by LTLS was 33%. The obtained cellulose product was creamy white. The extracted cellulose samples were characterized by using infrared spectroscopy (IR), thermogravimetry (TG), and X-ray diffraction (XRD). The results were consistent with that of standard cellulose. We confirmed that the LTLS extracted cellulose from corn straw with high purity.

## 1. Introduction

Cellulose is a natural polymer that has been used in paper making, medical treatment, clothing, and other applications [1,2,3,4,5]. Cellulose is mainly obtained from cotton, wood and other plants [6,7]. In recent years, with the proposal of the concept of the sustainable development of resources, the rational application of agricultural waste has become a hot topic for researchers [8,9]. Researchers have extracted cellulose from agricultural wastes, such as pineapple leaves, straw, bagasse, potato residue and soybean residue [10,11,12,13]. However, few reports have described the extraction of cellulose from corn straw. Approximately 1000 million tons of corn straw is produced annually worldwide, but the utilization rate of corn straw is less than 3% [6,7]. The extraction of cellulose from corn straw has been reported to improve the utilization rate of corn straw, prevent the waste of resources, and protect the environment [14,15,16].

The main components of corn straw are cellulose, hemicellulose, lignin, ash, lipids and other small molecules [17,18,19]. The cellulose content in corn straw is 30–40%, the hemicellulose content is 25–30%, and the lignin content is 10% [20]. Cellulose is present at the highest levels. Therefore, the extraction of cellulose from corn straw is feasible.

The systems used to extract cellulose from straw mainly include chemical systems, physical systems, biological systems and biochemical systems [21]. Wang and colleagues used different solution systems to extract cellulose, such as sodium hydroxide–acetic acid–sodium chlorite–acetone, nitric acid–sodium hydroxide, sodium hydroxide–sodium hypochlorite, and nitric acid–ethanol [22]. Based on these experimental results, cellulose is separated very well using the nitric acid-ethanol system. Nitric acid has strong volatility, oxidation and corrosiveness, which will damage the human body and environment. Waste liquor recovery is also more difficult. The commonly used physical system is high-speed agitation. Jiang and colleagues obtained nanocellulose from straw though high-speed agitation [23]. The cellulose obtained using the physical separation system is not pure. According to Bajpai et al., laccase selectively degrades lignin. Laccase and xylanase synergistically and effectively degrade lignin [24].

Chemical and biochemical systems efficiently remove lignin and hemicellulose from straw [22,25,26]. However, these two systems have some limitations, such as a high reaction temperature, high solvent pollution and an inability to recycle the used solutions. Studies aiming to analyze cellulose extraction should be performed and have goals toward the development of a low cost system that provides environmental protection and solvent recycling capabilities.

A new system for extracting cellulose from corn straw has been developed in this paper. The new system was named the low temperature laccase system (LTLS) and obtained highly pure cellulose. The organic acid solvent used in LTLS can be used at least eight times, reducing the waste liquid discharge. Laccase is a protein, and the waste liquid can be absorbed directly by the natural environment. Therefore, we consider LTLS an environmentally-friendly extraction system.

## 2. Experimental Procedures

**Materials:** All solvents were analytically pure reagents. All chemicals were purchased from Shanghai Darui Finechem Ltd (Shanghai, China). Corn straw was obtained from farm of Qiqihar City, Hei Longjiang Province, China (Qiqihar, China). Laccase (EC1.30.2) comes from Aspergillus oryzae, purchased from Anhui kuer biological engineering co. LTD. The optimum temperature for laccase is 55 °C and the optimum pH is 4.5. Standard cellulose was purchased from Aladdin Chemical Co., Ltd. (Shanghai, China), particle size was 25 µm, crystallinity was 91%.

**Methods:** Cellulose extraction from corn straw by the high temperature system (HTS): Corn straw powder (2 g) was placed in a Soxhlet extractor. A 2:1 ethanol:toluene ratio (60 mL:30 mL) was used as the circulating solvent and applied at 80 °C for 21 h to wash and dry the corn straw powder. The powder was placed in a reaction flask with NaClO (27 mL, 1.43 mol/L), water (3 mL) and glacial acetic acid (0.75 mL) and stirred at 80 °C for 2.5 h. Then, the corn straw powder was filtered and washed with water. When the pH of the filter solution reached 7, the filter residue was placed in an oven and dried. Next, the dry filter residue was reacted with an 8% NaOH solution for 2 h at 80 °C. Cellulose was obtained after filtration [22].

Cellulose extraction from corn straw by the low temperature system (LTS): Corn straw powder (2 g) was stirred for 84 h in a 5% NaOH solution (80 mL) at 0 °C. The sediment was filtered and flushed until the pH of the filter solution was 7. The filter residue was dried at 65 °C to obtain a dry powder. The filter residue was immersed in deionized water for 2 h and then reacted with a mixture of formic acid, glacial acetic acid and deionized water (volume ratio of 3:5:2, total solvent volume 100 mL, this solvent can be recycled) for 2.5 h at 60 °C. The deposit was filtered, washed and dried again.

Cellulose extraction from corn straw by the low temperature laccase system (LTLS): Corn straw powder (2 g) was stirred for 72 h in a 5% NaOH solution (80 mL) at 0 °C. The sediment was filtered and flushed until the pH of the filter solution was 7. A pale yellow powder was obtained by drying the filtered residue at 65 °C. The powder was immersed in deionized water for 2 h and then reacted with laccase (1 g) for 72 h in phosphoric acid buffer solution (80 mL, 0.20 mol/L, pH = 4.5) at 55 °C. Then, the reacted precipitates were separated by centrifugation (2000 r/min). The new precipitates were dried at 65 °C for 2.5 h. The dried sediment was then reacted with formic acid, acetic acid and deionized water (volume ratio 3:5:2, total solvent volume 100 mL, this solvent can be recycled) for 2 h at 60 °C. The deposit was filtered and dried again.

**Instruments:** The subject used infrared spectroscopy (IR, Perkin-Elmer, MA, America; Spectrum One B IR spectrophotometer) to characterize functional group of the products of HTS, LTS, LTLS, and standard cellulose. The products and KBR were mixed evenly and ground in the ratio of 1:100 to get sample of IR. Then scanning three times to get spectra. Thermogravimetry (TG, TA Instruments, New Castle, DE, America; Q5000IRS) was used to characterize mass loss of the products of LTS, LTLS, and standard cellulose. A total of 2.14 mg of sample was tested three times in 10 °C /min under nitrogen to get TG spectra. X-ray diffraction (XRD, BRUKER AXS GMBH, Karlsruhe, Germany; D8 Advance) was used to characterize crystalline state of the products of LTS, LTLS, and standard cellulose. The scanning condition of XRD was as follows: Wavelength of X-ray is 1.5418 A, scanning speed is 10°/min, scanning mode is 2-theta/theta, scanning type is continuous scanning. Calculation of crystallinity: The 2-theta/theta angle is 5° to 50°, the crystallinity (%) = peak area of the crystalline part / total area of scanning area peak × 100.

## 3. Results and Discussion 

### 3.1. Yield and Color of Various System Products

The high temperature system (HTS) that operates above 80 °C is a traditional method for extracting cellulose from plant straw [22]. The HTS produces cellulose at a high yield (42.8%) and high purity (Table 1, No. 1). The purified cellulose is white, similar to the color of standard cellulose. White indicates highly pure cellulose. However, the HTS has three limitations: A higher reaction temperature, the use of toxic reagents and chlorine production. Therefore, we introduced a new system, the low temperature system (LTS). When the concentration of the NaOH solution was 5% (Table 1, No. 3), the product obtained using the LTS exhibited the whitest color. The purity of cellulose produced in the other two groups (Table 1, No. 2 and No. 4) was lower than the product obtained with the 5% NaOH solution, because the products were more yellow in color. We postulate that the repeated freezing and thawing of corn straw powder relaxes the macromolecule structure of the corn straw powder, promoting the dissolution of lignin in solvents. The LTS is more environmentally friendly than the HTS, because the LTS does not use toxic substances and the solvents can be recycled. However, the LTS is imperfect. The lignin in the LTS product was not completely removed because the color of the product was faint yellow. The LTS yield is low because a small amount of cellulose was dissolved in long-term low temperature reactions. Laccase was added to the LTS to solve the problem that lignin was not completely removed. The amount of dissolved cellulose was also reduced by decreasing the reaction time at low temperature. We named the new system the low temperature laccase system (LTLS), which yielded a creamy white product, indicating that the product was almost completely free of lignin. The product yield increased to 33% after decreasing the reaction time. The optimal input was 1 g of laccase per 2 g of corn straw powder, because the yield did not increase when the laccase concentration exceeded 1 g (Table 1, No. 6). Laccase catalyzes the single-electron oxidation of phenols and aromatic amines, while reducing oxygen molecules to water. The ester bond between lignin and hemicellulose is destroyed by laccase and reduced to a hydroxyl group [27]. Laccase is a nontoxic protein that is decomposed by the natural environment. The product yield and purity of the LTLS were better than the LTS. In addition, the LTLS was better than the HTS in terms of environmental protection.

### 3.2. Infrared Analysis

**Figure 1:**
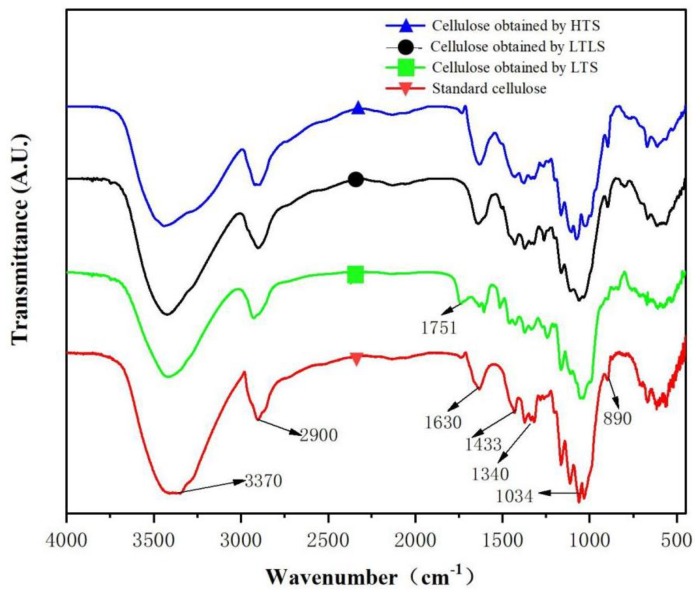
Infrared spectra of cellulose obtained using the LTS, LTLS, HTS, and standard cellulose.

Infrared spectra of standard cellulose and other samples obtained using the LTLS, LTS and HTS are shown in Figure 1. The red curve is the infrared image of standard cellulose. The absorption peak at 3370 cm^−1^ is attributed to the stretching vibration of –OH in cellulose. The absorption peak at 2900 cm^−1^ represents the stretching vibration of CH in cellulose. 1640cm^−1^ is the O–H bending vibration caused by the hydrogen bonding of water in cellulose, indicating that standard cellulose contains water [28]. The absorption peak at 1340 cm^−1^ is the stretching vibration peak of –CH_2_ in cellulose. The absorption peak at 1034 cm^−1^ is the stretching vibration of C–O–C in cellulose. The bending vibration of C–H in cellulose produces an absorption peak at 890 cm^−1^, which is a special absorption peak of cellulose-I structure [29]. The products of HTS and the LTLS are the same as the characteristic peaks detected in the infrared spectrum of standard cellulose. LTLS performed under low temperature reaction conditions effectively extracted cellulose from corn straw at high purity. The LTS product showed an absorption peak at 1751 cm^−1^, which is a characteristic peak of the stretching vibration of the nonconjugated C=O in lignin [30]. Thus, the LTS product contains lignin. However, the cellulose obtained using the LTLS did not show a new absorption peak at 1751 cm^−1^, indicating that the introduction of laccase successfully removed the lignin that remained after extraction with the LTS.

### 3.3. TG Analysis

**Figure 2:**
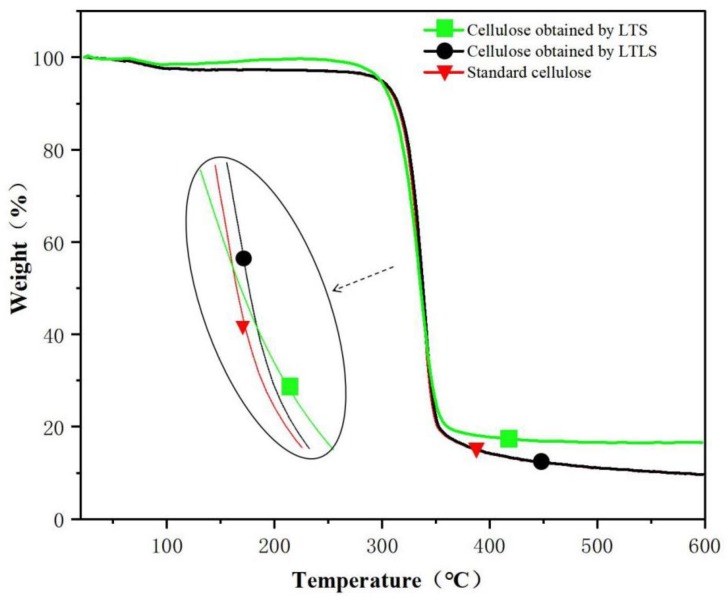
TG curves of LTS and LTLS products and standard cellulose.

As shown in the TG spectra (Figure 2) standard cellulose evaporated water at 100 °C The cellulose decomposed quickly at 300 °C and decomposed completely at 500 °C. The final sample contained 8% ash. The curve of the product extracted using LTLS is the same as standard cellulose. The ash content of the LTS product after combustion was 20%, which is higher than standard cellulose, indicating that the LTS product contained more lignin. The benzene ring structure in lignin is not completely burned at 600 °C; therefore, the end of the TG curve of the LTS product shows a large amount of remaining ash. After the addition of laccase, the ash content in the LTLS product was significantly lower than the LTS product. The TG curve of the LTLS product was the same as the standard cellulose sample, and the residual amount of ash was 10%. We concluded that the introduction of laccase effectively removes lignin from the corn straw, increasing the purity of the extracted cellulose. The LTLS successfully extracts high-purity cellulose under low temperature conditions at a high yield.

### 3.4. X-ray Diffraction

**Figure 3:**
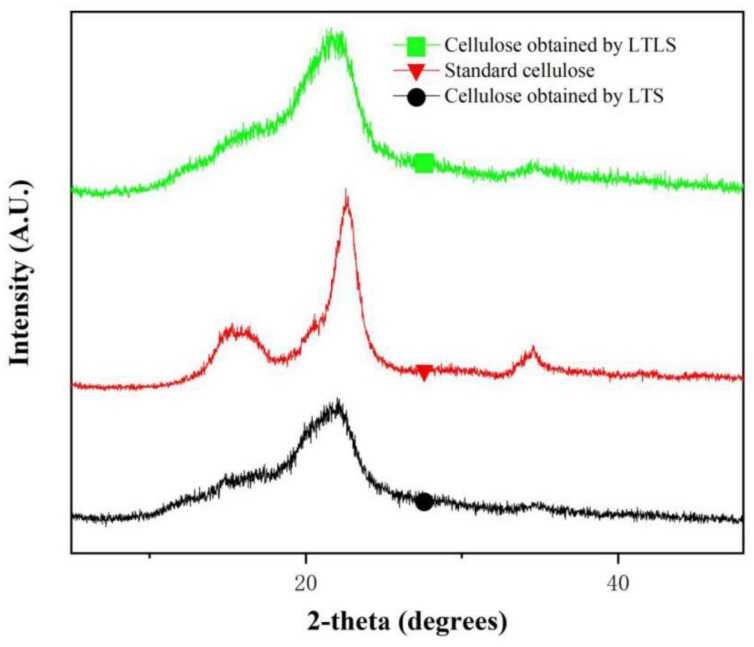
XRD spectrum of LTS and LTLS products and standard cellulose (wavelength: 1.5418 A).

As shown in the XRD spectrum (Figure 3), the products extracted using LTS and LTLS have the same crystalline region as standard cellulose. The standard cellulose has obvious peaks at 15°, 16.9°, 23° and 34°. These four peaks respectively correspond to the 110, 101, 200 and 004 crystal faces of natural cellulose-I [31]. These four peaks were observed at the same positions for the products extracted by using LTS and LTLS, indicating that the crystalline structures of both products were same as standard cellulose. The relative crystallinity of products obtained using LTS was 76.5% and products obtained using LTLS was 88%. The low crystallinity of products obtained using LTS is due to the hydrolysis of a small amount of cellulose by organic acid solutions. The organic acid solution hydrolyzes the polyarabinose and glucose in the hemicellulose, and hydrolyzes the lower or incompletely crystallized glucose in the cellulose [32,33]. The decrease in diffraction intensity is also related to the hydrolysis of cellulose by organic acid solution. When laccase is added, the crystallinity of cellulose increases significantly. A potential explanation is that laccase facilitates the easy removal of hemicellulose and lignin that would result in easy accessibility of organic acid in the cellulose chains and dissolve the amorphous domains. The LTLS is characterized by a high yield and purity, and it maximizes the retention of the cellulose-I structure of natural cellulose. The LTLS product has a relatively high degree of crystallinity, making it easier to process and use.

### 3.5. Effects of Circulating Organic Acids on the Yield of the LTS and LTLS

**Figure 4:**
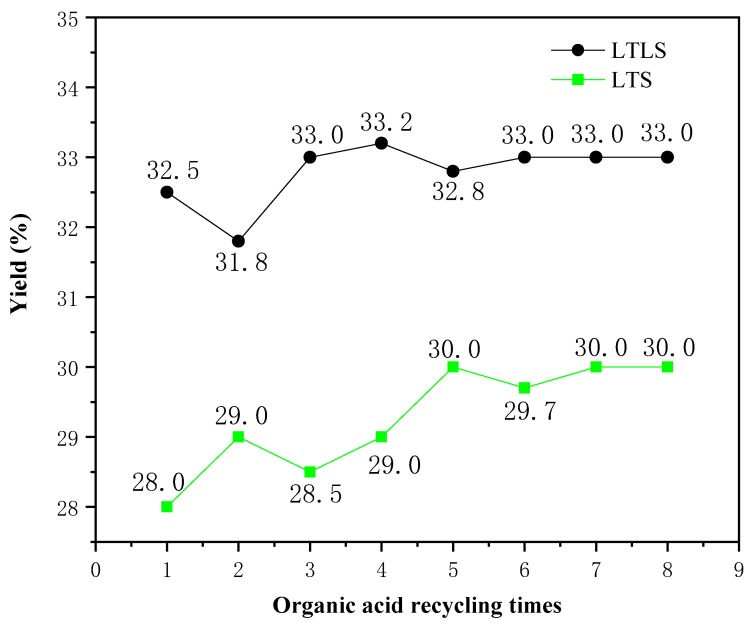
Effects of circulating organic acids on the yield of the LTS and LTLS.

The effect of recycling the mixed organic acid solution on the yield was studied. The relationship between the number of recycles and the yield is shown in Figure 4. The yields of LTLS and LTS increased as the recycle number increased, due to the weakening ability of the organic acid mixture to hydrolyze cellulose. The yields of LTLS and LTS increased significantly when the mixed organic acid solution was used one to four times, because the mixed organic acid solution hydrolyzed some of the incompletely crystallized cellulose. When the mixed organic acid solution was used four times, the yields of LTS and LTLS both reached a stable value, indicating that the organic acid mixture had optimally removed the hemicellulose. Based on these results, the effect of recycling the mixed organic acid solution on the yield is small, confirming that the recycling the mixed organic acid solution is feasible. Moreover, the experiment in which the solution was recycled eight times reduced at least 800 mL of waste liquid discharge, reducing the pressure of waste liquid treatment on cellulose extraction. Finally, we also recycled and reused the mixed organic acid solution by distillation to result in zero waste liquid discharge. Thus, we propose that LTLS is a new, environmentally-friendlysystem for extracting cellulose from corn straw.

## 4. Conclusions

A new system for extracting cellulose from corn straw was developed successfully in this paper, namely, the LTLS. The characteristic peaks of the products observed in IR, TG and XRD spectra were consistent with standard cellulose. The yield of cellulose obtained using the LTLS was 33%. The LTLS has the advantages of a good biological affinity, high-yield crystallizability and easy operation. The LTLS was operated in a low temperature environment and thus solved the problem that cellulose in corn straw could previously only be extracted with a high temperature system. 

## Figures and Tables

**Table 1:** Yield and color of products obtained using HTS, LTS and LTLS.

System	No	NaOCl (mol/L)	NaOH (%)	Laccase (g)	T/°C	Time/h	Yield ^a^/%	Color
HTS	1	1.43	-	-	80	2.5	42.8	white
LTS	2	-	5.0	-	80	84	28.0	darker yellow
	3	-	5.0	-	0	84	30.0	faint yellow
	4	-	7.0	-	0	84	35.5	darker yellow
LTLS	5	-	5.0	0.70	0	72	29.6	faint yellow
	6	-	5.0	1.00	0	72	33.0	creamy white
	7	-	5.0	1.50	0	72	33.0	creamy white

^a^ This refers to efficiency of the entire process.
